# Plain Language Summary of Publication: Design of the Phase 3 FREESIA-1 and FREESIA-3 Trials of Nipocalimab in Fetal and Neonatal Alloimmune Thrombocytopenia

**DOI:** 10.1055/a-2761-1775

**Published:** 2025-12-29

**Authors:** Pamela Baker, Babajide Keshinro, Barbara Stegmann, Abbie Oey, Cathye Shu, Dirk Heerwegh, Rebecca Zaha, Hillary Van Valkenburgh

**Affiliations:** 1 Johnson & Johnson, Spring House, Pennsylvania, United States; 2 Johnson & Johnson Biologics BV, Leiden, The Netherlands; 3 Johnson & Johnson, Beerse, Belgium

**Keywords:** FNAIT, study design, efficacy, safety, nipocalimab

## Abstract

This article is a plain language summary of publication (PLSP) of the following articles: “Design of a Phase 3, Multicenter, Randomized, Placebo-Controlled, Double-Blind Study of Nipocalimab in Pregnancies at Risk for Fetal and Neonatal Alloimmune Thrombocytopenia (FREESIA-1)” by Tiller et al published in *American Journal of Perinatology* on July 28, 2025 (doi:10.1055/a-2666-5642) and “Design of a Phase 3, Multicenter, Randomized, Open-Label Study of Nipocalimab or IVIG and Prednisone in Pregnancies at Risk for Fetal and Neonatal Alloimmune Thrombocytopenia (FREESIA-3)” by Bussel et al published in *American Journal of Perinatology* on November 25, 2025 (doi: 10.1055/a-2753-9323). This PLSP describes the design of the phase 3, placebo-controlled FREESIA-1 and open-label FREESIA-3 trials, which will help researchers understand if nipocalimab, an investigational treatment, can be used to safely treat pregnant individuals who are at risk for fetal and neonatal alloimmune thrombocytopenia (FNAIT). This PLSP will help the general public, including those affected by FNAIT, and health care professionals understand the two studies. It provides information on the eligibility criteria, study design, treatments, and outcomes of interest. An infographic summary of this article is available in the Supplementary Material (available in the online version only).

## Synopsis

### What Is This Summary About?

This is a summary of two scientific publications that describe the design of the placebo-controlled FREESIA-1 and open-label FREESIA-3 phase 3 clinical trials in pregnant participants at risk for fetal and neonatal alloimmune thrombocytopenia (FNAIT). In these studies, researchers will determine if an investigational treatment called nipocalimab can be used safely and effectively to treat pregnant individuals who are at risk for FNAIT.

### What Happens in These Studies?

FREESIA-1 is a randomized, placebo-controlled, double-blind, phase 3 trial of nipocalimab in pregnant participants at risk for FNAIT who have had a previous FNAIT-affected pregnancy without an intracranial hemorrhage (ICH; bleeding in the brain) or severe bleeding in the fetus or baby (known as “standard-risk” FNAIT). About 39 participants will take part in the study and will be randomly split into two groups. For every three participants, two will receive nipocalimab and one will receive a placebo, which is a treatment that does not have any active medicine. Every participant will receive their assigned weekly treatment infusions through a vein. By including a placebo group, researchers can assess whether the observed treatment effects in the participants and their children are caused by nipocalimab. FREESIA-1 will be conducted at study centers located in Belgium, Brazil, France, Hungary, Israel, Italy, Norway, Slovakia, Slovenia, Spain, Sweden, and Switzerland, with planned sites in additional countries. ^1^

FREESIA-3 is a randomized, open-label, phase 3 trial of nipocalimab or intravenous immunoglobulin (IVIG; a type of antibody treatment) with prednisone (a type of steroid hormone), which is the standard treatment for pregnancies at risk for FNAIT in most countries. The FREESIA-3 study will first include pregnant participants with “standard-risk” FNAIT. Later, it will include participants with a baby who experienced ICH or severe bleeding in a prior FNAIT-affected pregnancy (known as “high-risk” FNAIT). About 50 participants will be randomly split into two groups. For every five participants, four will receive weekly treatment with nipocalimab and one will receive weekly treatment with IVIG and prednisone. The groups will also be based on the type of blood incompatibility they have (either human platelet antigen [HPA]-1a or HPA-5b). Every participant will receive their assigned weekly treatment infusions through a vein. FREESIA-3 will be conducted at study centers in Austria, Germany, Poland, the Netherlands, the United Kingdom, and the United States. ^2^

In both studies, pregnant participants will have weekly visits to the study centers to receive study treatment. Babies will be monitored every 2 weeks through prenatal ultrasounds to see how they are growing/developing and to look for any signs of bleeding. After delivery, maternal participants will be monitored for 6 months and babies for 2 years.

### What Key Results Will the Studies Provide?

Both studies will look at the same clinical outcomes (known as “endpoints”) to understand how well nipocalimab works. These studies will evaluate how many fetuses or babies do not survive, have major bleeding, or are born with a very low number of platelets (small blood cells that are essential to help stop bleeding).

In FREESIA-1, the results will be compared between the nipocalimab and placebo groups, while FREESIA-3 will look at how treatment with nipocalimab or IVIG with prednisone works. To identify any side effects that may be caused by nipocalimab treatment, maternal participants will be monitored during pregnancy and for 6 months after giving birth, and babies will be monitored for the first 2 years of life. All side effects will be recorded.

### Who Is This Summary For?

This summary is meant to help the general public, especially individuals and families affected by FNAIT, better understand the studies. It may also be useful for doctors, nurses, and other health care providers. More detailed information and references can be found in the original articles. 1 2 Links to the original articles can be found at the end of this summary.

### Who Sponsored the Studies?

These studies are sponsored by Johnson & Johnson.

## Content

### What Is Fetal and Neonatal Alloimmune Thrombocytopenia?

**Fig. 1:**
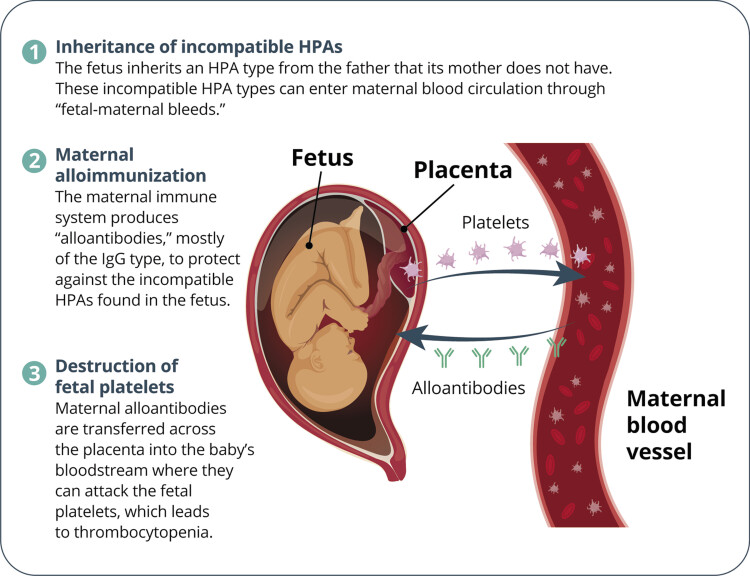
Development of FNAIT.

FNAIT is a rare pregnancy complication that occurs when a pregnant individual's immune system reacts against the fetal platelets due to genetically different, paternally derived HPAs (naturally occurring proteins that are found on the surface of platelets; Fig. 1 ). In response to the mismatch between HPAs, immunoglobulin G (IgG) alloantibodies are produced by the pregnant individual's immune system. These alloantibodies then move across the placenta and cause fetal platelets to be destroyed, which leads to a condition called thrombocytopenia (lower-than-normal number of platelets) in the fetus and baby. There are several types of HPAs that can contribute to FNAIT, but the most common one is HPA-1a, followed by HPA-5b.

FNAIT can be very serious. If the fetus or baby has bleeding in the brain (called an ICH), it can potentially lead to death or long-term brain damage. Pregnancies can be classified as either standard-risk or high-risk. Standard-risk means the pregnant individual had a previous pregnancy affected by FNAIT, but the fetus or baby did not have an ICH or severe bleeding. High-risk means the pregnant individual had a previous pregnancy affected by FNAIT with ICH or severe bleeding in the fetus or baby. The risk of ICH is lower in standard-risk versus high-risk pregnancies. Furthermore, following the birth of a baby affected by FNAIT, there is a high likelihood (up to 90%) that FNAIT may happen again in a future pregnancy.

### How Is a Pregnancy at Risk of FNAIT Treated Now?

**Fig. 2:**
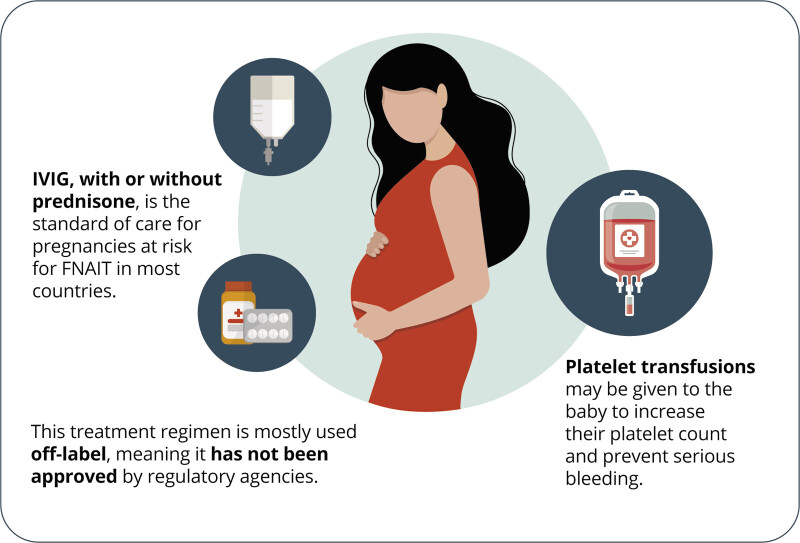
Current management of pregnancies at risk of FNAIT.

There are currently no approved treatments for pregnancies in either the standard-risk or high-risk FNAIT groups. Off-label administration of IVIG, with or without prednisone, is considered to be the standard of care for the antenatal treatment of FNAIT in most countries because it has been associated with a lower risk of ICH when used for high-risk FNAIT pregnancies ( Fig. 2 ). However, use of these treatments can vary in different parts of the world. Additionally, treatment with IVIG and prednisone may put a strain on patients due to their side effects, as well as the cost, treatment access, and administration time needed for IVIG. Platelet transfusions may be given to babies with very low platelet counts after birth to reduce the risk of life-threatening complications.

### What Is Nipocalimab?

Nipocalimab is a once-weekly treatment administered through a vein that is being studied in pregnancies at risk for FNAIT, and it is the only treatment currently being tested in phase 3 studies for this condition. Nipocalimab is a type of antibody that blocks the neonatal Fc receptor (FcRn), which is a protein found on cells in the placenta that can move IgG alloantibodies from the maternal to fetal bloodstream. FcRn works like a recycling system to keep high levels of maternal IgG in the bloodstream. By blocking FcRn, nipocalimab helps lower the levels of maternal IgG, including alloantibodies, that are available for transfer to the fetus.

Nipocalimab is also under clinical development for the prevention of severe hemolytic disease of the fetus and newborn (HDFN), a similar IgG alloantibody–mediated pregnancy condition. In a recent phase 2 study that involved pregnant participants with early-onset severe HDFN (UNITY; *ClinicalTrials.gov* Identifier: NCT03842189), nipocalimab was able to prevent or delay fetal anemia and reduce the need for intrauterine transfusions of red blood cells, with manageable side effects. The most frequently reported side effects were those expected due to HDFN, pregnancy, or preterm birth and were not necessarily related to nipocalimab. These positive findings support further evaluation of nipocalimab for the treatment of FNAIT in the FREESIA-1 and FREESIA-3 studies.

### Why Are the Two Phase 3 FREESIA-1 and FREESIA-3 Studies Being Conducted?

When a new medication is being tested, phase 3 trials help to provide the data needed to support its approval for use. Researchers use these carefully designed studies to ensure a new medication is safe and works effectively to treat specific conditions. Typically, participants who receive the new drug are compared with another group that receives either a placebo (inactive treatment; known as “placebo-controlled” studies), to see whether the observed effects are caused by the treatment, or an established treatment, to assess if the new treatment offers additional benefits relative to the currently available treatment. The studies are also “randomized,” which means that a computer program is used to randomly assign the treatment that each participant receives. Studies can also be “double-blind,” which means that neither the researchers nor participants will know which treatment they are receiving until the study ends, or “open-label,” which means that both the researchers and participants know which medication is being given.

There are two separate studies that are evaluating nipocalimab for the prevention of FNAIT. FREESIA-1 is a randomized, placebo-controlled, double-blind, phase 3 study that is designed to evaluate the efficacy and safety of nipocalimab in pregnant participants at standard-risk for FNAIT. FREESIA-1 uses a placebo group as the control to reflect the way FNAIT has been managed in Norway for decades. This comparator will allow researchers to establish the frequency and magnitude of changes that occur in the absence of a clinical intervention. ^1^

FREESIA-3 is a randomized, open-label, phase 3 study that will assess the efficacy and safety of nipocalimab or IVIG with prednisone in pregnant participants at standard- and high-risk for FNAIT. FREESIA-3 uses IVIG with prednisone as the reference arm to align with the standard of care in most countries. ^2^

An infographic summary of this article is available in Supplementary Material (available in the online version only).

### Where Will FREESIA-1 Take Place, and Who Will Take Part in the Study?

**Fig. 3:**
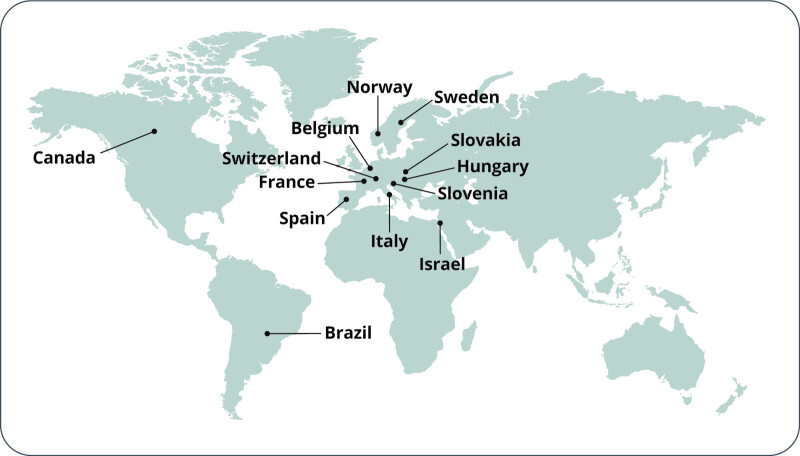
Location of participating centers in the FREESIA-1 study. (For more information on FREESIA-1 study sites, please visit https://www.clinicaltrials.gov/study/NCT06449651 )

**Fig. 4:**
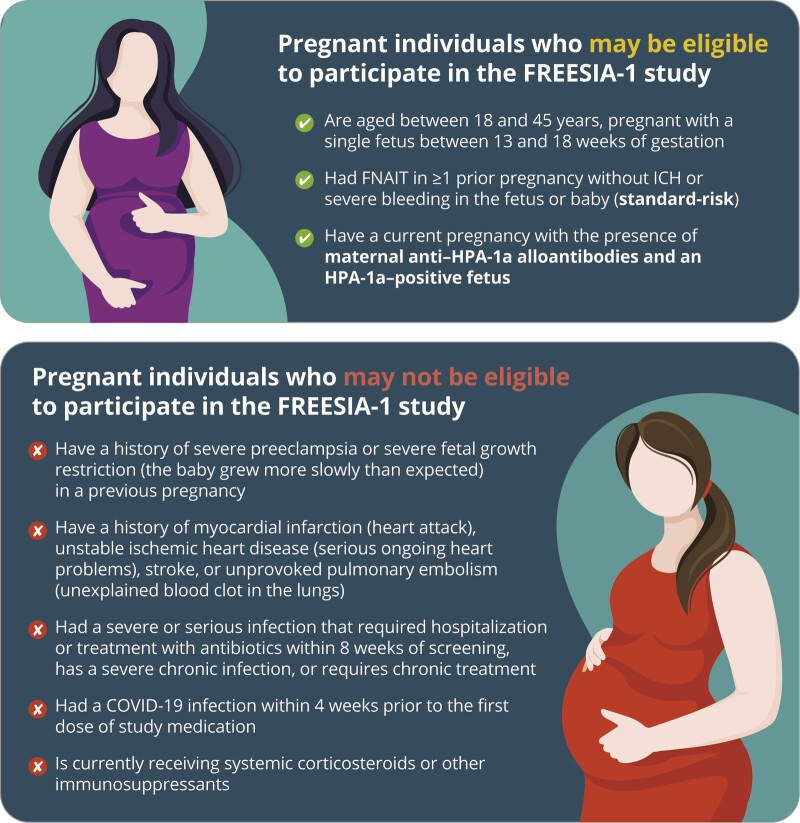
Select eligibility criteria for the FREESIA-1 study. (Complete eligibility criteria can be found in the original article or by visiting https://www.clinicaltrials.gov/study/NCT06449651 )

FREESIA-1 is enrolling approximately 39 HPA-1a–alloimmunized pregnant individuals at standard-risk for FNAIT. Approximately 20 maternal-fetal medicine centers worldwide will take part in the FREESIA-1 trial ( Fig. 3 ). To be eligible for the study, maternal participants must meet the criteria presented in Fig. 4 .

### How Will the FREESIA-1 Study Be Carried Out?

**Fig. 5:**
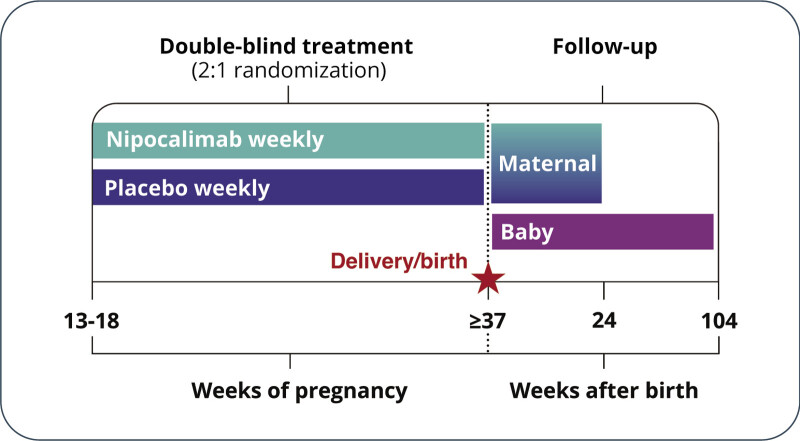
Study design of FREESIA-1.

In the FREESIA-1 study, two-thirds of the participants will receive nipocalimab and one-third will receive placebo ( Fig. 5 ). Both groups will receive weekly infusions of their assigned treatment through a vein from the time they enter the study (between 13 and 18 weeks of gestation) until delivery. Pregnant participants will have visits at the study centers every 2 weeks for prenatal ultrasounds to monitor the babies' growth/development and to look for any signs of bleeding. After birth, babies will be cared for and a cranial ultrasound and platelet count will be checked.

### Where Will FREESIA-3 Take Place, and Who Will Take Part in the Study?

**Fig. 6:**
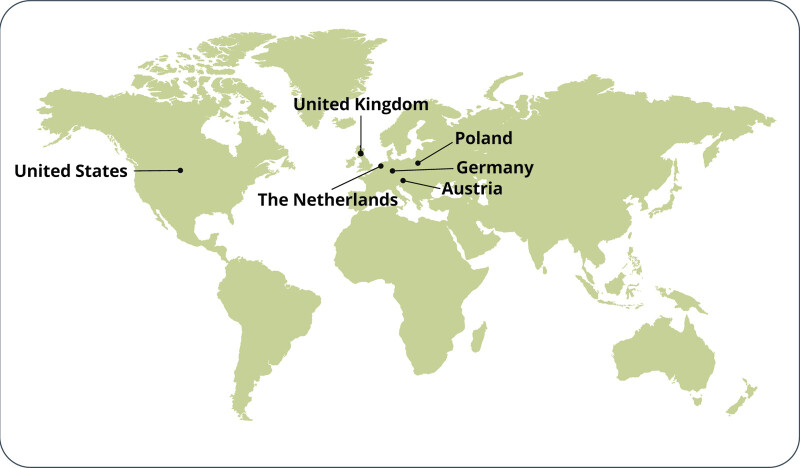
Location of participating centers in the FREESIA-3 study. (For more information on FREESIA-3 study sites, please visit https://www.clinicaltrials.gov/study/NCT06533098 )

**Fig. 7:**
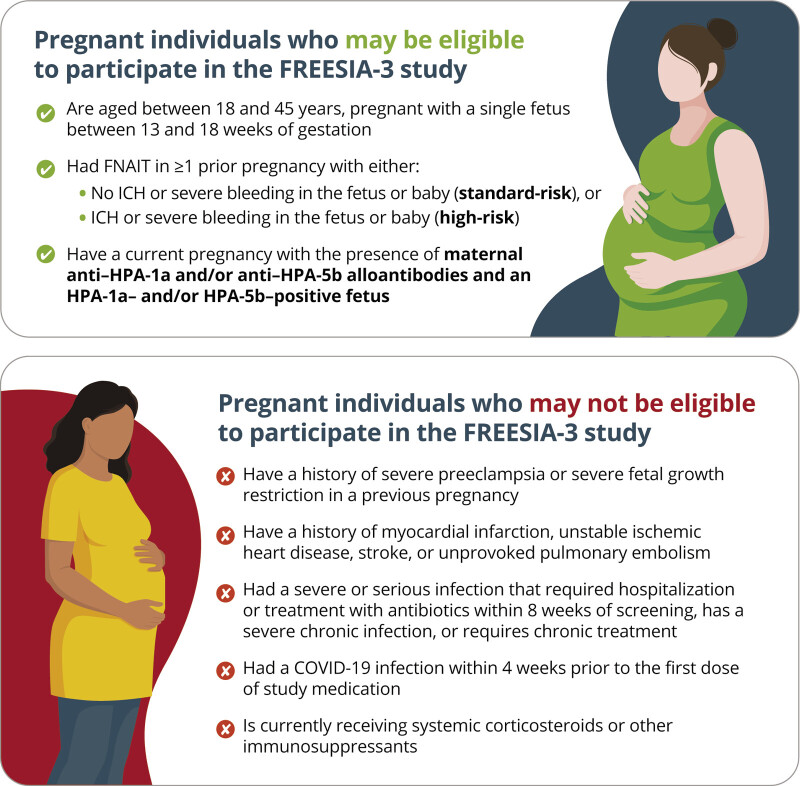
Select eligibility criteria for the FREESIA-3 study. (Complete eligibility criteria can be found in the original article or by visiting https://www.clinicaltrials.gov/study/NCT06533098 )

The FREESIA-3 study is enrolling approximately 50 HPA-1a–alloimmunized and/or HPA-5b–alloimmunized pregnant individuals who are at standard-risk or high-risk for FNAIT. Approximately 35 global centers will be involved in the FREESIA-3 trial ( Fig. 6 ). To be eligible for the study, maternal participants must meet the criteria presented in Fig. 7 .

### How Will the FREESIA-3 Study Be Carried Out?

**Fig. 8:**
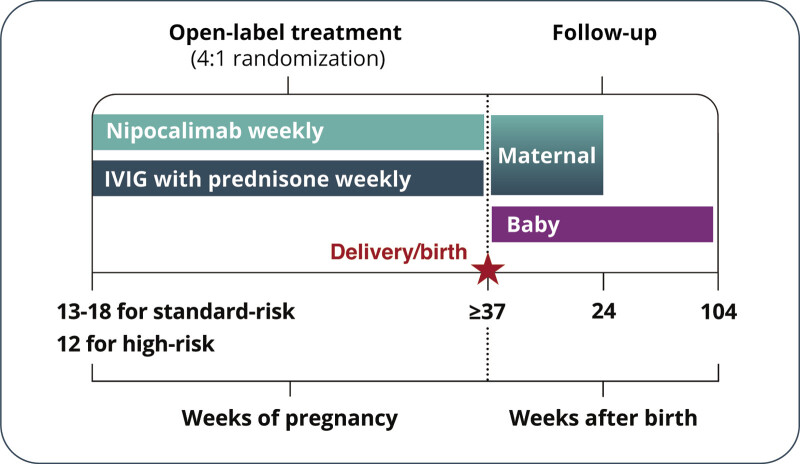
Study design of FREESIA-3.

In the FREESIA-3 study, participants will first be grouped into two cohorts based on the type of blood incompatibility they have, either HPA-1a or HPA-5b ( Fig. 8 ). For every five participants, four will receive nipocalimab and one will receive IVIG with prednisone. All participants will receive each assigned treatment once weekly. Those who receive nipocalimab will receive treatment as an infusion through a vein from the time they enter the study (between 13 and 18 weeks of gestation) until delivery. IVIG with prednisone doses will depend on whether the pregnancy is considered standard-risk or high-risk for FNAIT and will account for the gestational age of the fetus.

### How Will Researchers Measure the Efficacy of Nipocalimab?

**Fig. 9:**
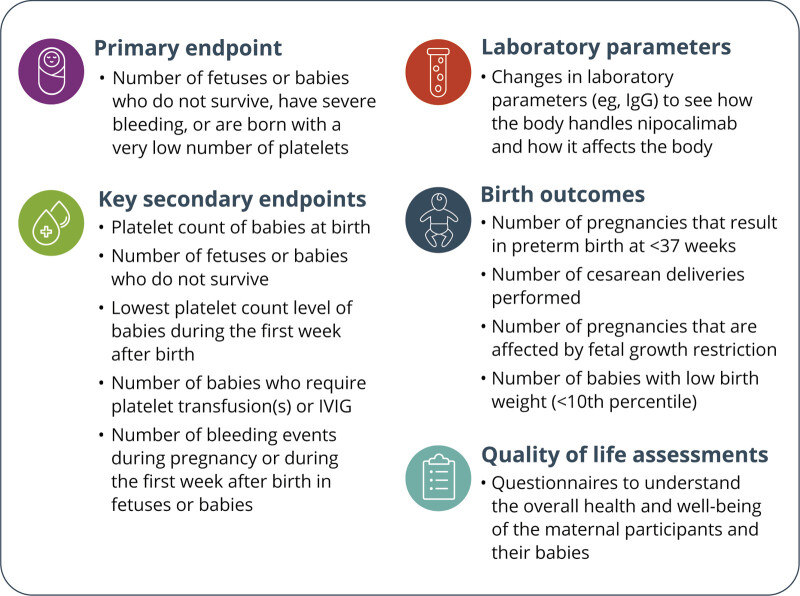
Select efficacy and safety assessments in FREESIA-1 and FREESIA-3. (Complete efficacy assessments can be found in the original articles or by visiting https://www.clinicaltrials.gov/study/NCT06449651 and https://www.clinicaltrials.gov/study/NCT06533098 )

The efficacy of nipocalimab will be measured by assessing a variety of clinical outcomes, which are the same for both studies. The main outcome, or “primary endpoint,” will evaluate the number of fetuses or babies who do not survive, have severe bleeding, or are born with a very low number of platelets ( Fig. 9 ). This primary endpoint is the most important result that will help researchers determine the efficacy of nipocalimab. In FREESIA-1, the results will be compared between those who received nipocalimab and placebo. In FREESIA-3, the results will be reported for those who received nipocalimab or IVIG with prednisone.

Researchers will also measure other clinical outcomes and ask participants and caregivers to complete questionnaires that are related to their health and that of their babies. Fig. 9 shows key assessments for both studies that are related to efficacy and will be measured during pregnancy and after birth in maternal participants and their babies.

### How Will Researchers Monitor the Safety of Nipocalimab?

Side effects in pregnant participants and liveborn babies will be monitored and recorded whether they are related to the treatment they received or not. In maternal participants, any reactions that occur during pregnancy and during the 6 months after birth will be recorded. In babies, any reactions, such as infections, bleeding, or low levels of IgG, that occur during the first 2 years of life will be recorded. Additionally, development will be assessed using the Bayley Scales of Infant and Toddler Development, a standardized tool to learn how babies are developing in various key areas.

### What Impact Will the Results of the FREESIA-1 and FREESIA-3 Studies Have?

Results from the placebo-controlled FREESIA-1 study and the open-label FREESIA-3 study will provide the necessary data to support the approval of nipocalimab for use in pregnancies at risk for FNAIT. The findings of these studies may support future clinical research on nipocalimab for other pregnancy-related immune conditions.

### Where Can Readers Find More Information on These Studies?

**You can read the original FREESIA-1 article here:**

The complete bibliography of the FREESIA-1 study is present in the reference. ^1^ The FREESIA-1 trial started on November 11, 2024, and is expected to end on December 11, 2029.

You can read more about the FREESIA-1 study by visiting *https://www.clinicaltrials.gov/study/NCT06449651* . If you are interested in participating in the study or have questions about the study or nipocalimab, please visit *https://clinicaltrials.jnj.com/en/study-detail/NCT06449651* .

**You can read the original FREESIA-3 article here:**

The complete bibliography of the FREESIA-3 study is present in the reference. ^2^ The FREESIA-3 trial started on February 10, 2025, and is expected to end on December 5, 2029.

You can read more about the FREESIA-3 study by visiting *https://www.clinicaltrials.gov/study/NCT06533098* . If you are interested in participating in the study or have questions about the study or nipocalimab, please visit *https://clinicaltrials.jnj.com/en/study-detail/NCT06533098* .

More general information on clinical studies can be found at *https://www.clinicaltrials.gov/ct2/about-studies/learn* .
