# Supplementary material for: Plain Language Summary of Publication: Design of the Phase 3 FREESIA-1 and FREESIA-3 Trials of Nipocalimab in Fetal and Neonatal Alloimmune Thrombocytopenia
Source: Am J Perinatol. 2025 Dec 29;43(9):1256–63. doi: 10.1055/a-2761-1775 (PMC13275166; doi:10.1055/a-2761-1775)
Supplement: Supplementary file 1 — Supplementary Material [file 10-1055-a-2761-1775_27786406.pdf]

Two phase 3 trials are evaluating nipocalimab for the treatment of pregnancies at risk for fetal and neonatal alloimmune thrombocytopenia (FNAIT).

What is FNAIT?

- FNAIT occurs when the **human platelet antigens (HPAs) on platelets of a pregnant individual and their developing fetus are mismatched**. In response, **immunoglobulin G (IgG) alloantibodies** produced by the pregnant individual's immune system **cross the placenta and destroy fetal platelets**, causing thrombocytopenia (lower-than-normal number of platelets)
- **FNAIT can be life threatening** if intracranial hemorrhage (ICH; bleeding in the brain) or major bleeding into an organ occurs in the fetus or baby
- In pregnancies with a history of FNAIT without ICH or severe bleeding in the fetus or baby (**standard-risk FNAIT**), the risk of ICH is lower compared with pregnancies that have a history of FNAIT accompanied by ICH or severe bleeding (**high-risk FNAIT**)

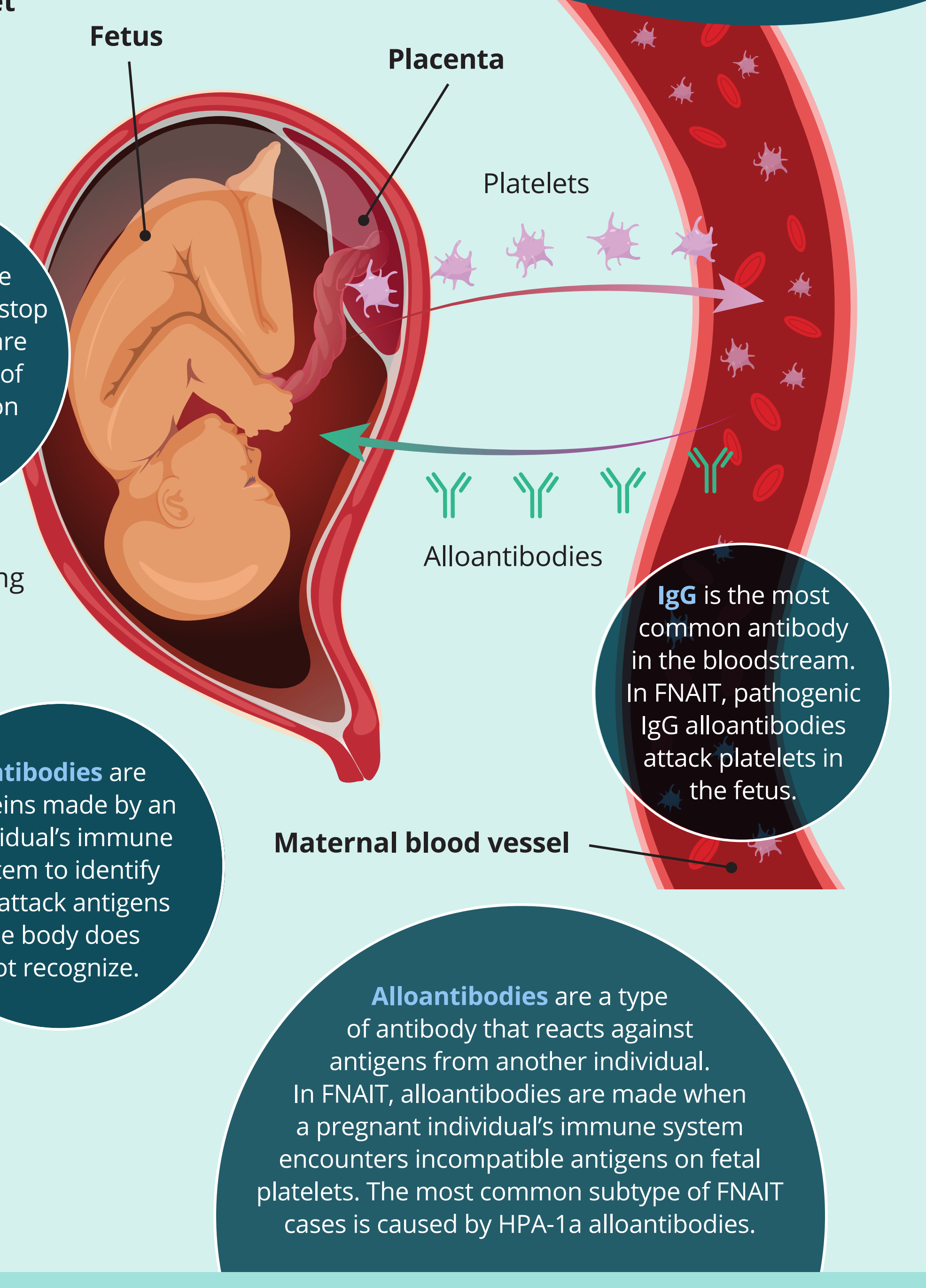

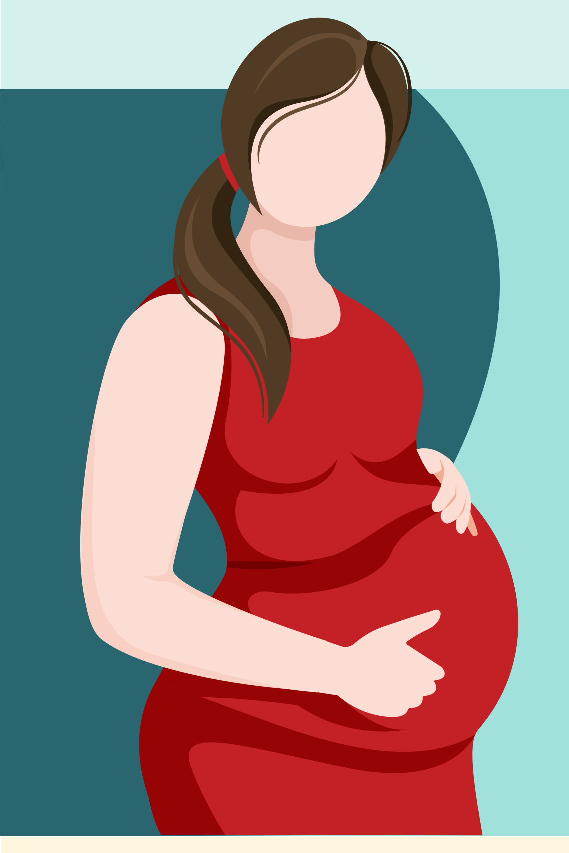

How is FNAIT treated now?

- There are currently **no approved treatments** for pregnancies at risk of FNAIT
- Off-label use of **intravenous immunoglobulin (IVIG), with or without prednisone**, is considered to be the **standard of care** for antenatal treatment of FNAIT in most countries; however, there are regional variations in how these treatments are used
- After birth, **platelet transfusions** may be given to babies with low platelet counts to reduce the risk of life-threatening complications

How does nipocalimab work?

- Nipocalimab is in **clinical development for FNAIT**
- Nipocalimab works by **binding to and blocking the neonatal Fc receptor (FcRn), which is the only transporter allowing IgG to cross the placenta** and transfer IgG to the fetus*
- In a recent phase 2 study, nipocalimab **delayed or prevented fetal anemia and the need for intrauterine transfusions** with an acceptable safety profile in pregnancies at high risk for early-onset severe hemolytic disease of the fetus and newborn (HDFN), suggesting it may be a potential treatment in other IgG alloantibody–mediated perinatal diseases, such as FNAIT

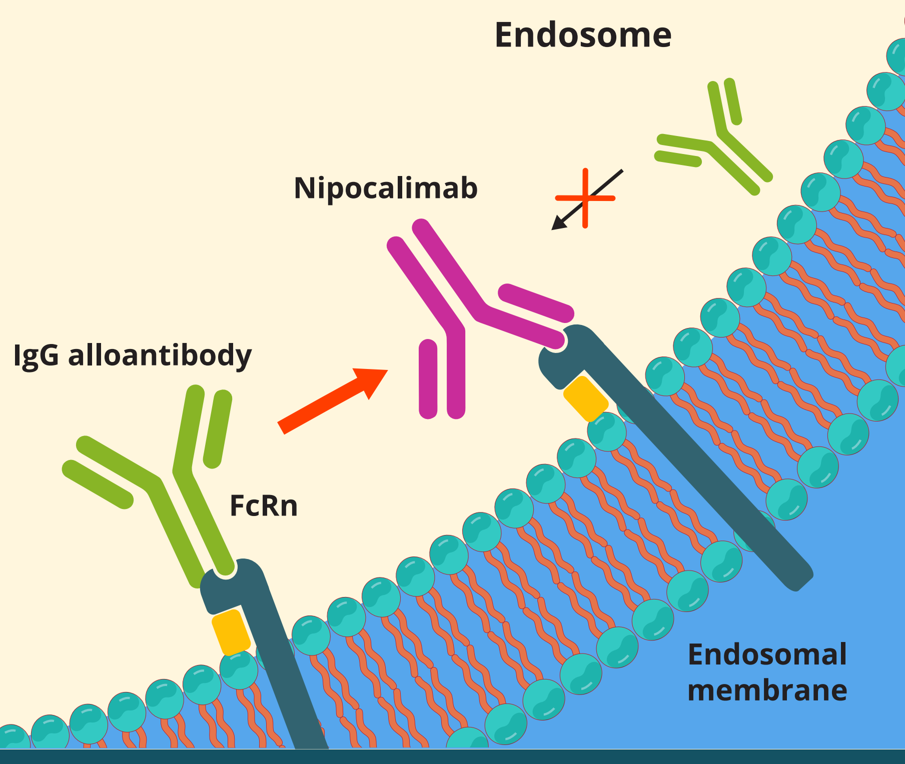

Two randomized, global, multicenter, phase 3 studies, FREESIA-1 and FREESIA-3, are planned to evaluate the safety and efficacy of nipocalimab in pregnancies at risk for FNAIT.

FREESIA-1 is a placebo-controlled, double-blind study of nipocalimab.

Study population

Pregnant individuals aged 18 to 45 years who:

- Are carrying 1 developing fetus between 13 and 18 weeks of gestation
- Have a history of ≥1 previous pregnancy affected by FNAIT without ICH or severe bleeding (**standard-risk**)
- Have **maternal anti–HPA-1a alloantibodies and an HPA-1a–positive fetus** in the current pregnancy

Study design

- Participants (N ≈ 39) are randomly assigned in a 2:1 ratio to receive weekly intravenous **nipocalimab or placebo**
- During the treatment period, participants will have **ultrasound monitoring every 2 weeks**
- After birth, babies will receive a cranial ultrasound scan and a platelet count assessment
- Follow-up will be 6 months after delivery for maternal participants and 2 years for babies
- Study sites are located in Belgium, Brazil, France, Hungary, Israel, Italy, Norway, Slovakia, Slovenia, Spain, Sweden, and Switzerland, with sites planned in additional countries

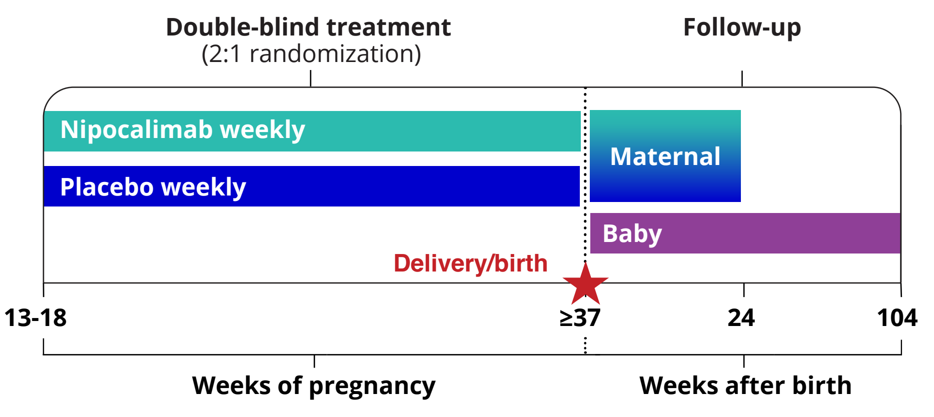

FREESIA-3 is an open-label study of nipocalimab or IVIG with prednisone.

Study population

Pregnant individuals aged 18 to 45 years who:

- Are carrying 1 developing fetus between 13 and 18 weeks (**standard-risk**; without ICH or severe bleeding in ≥1 prior FNAIT pregnancy) or 12 weeks of gestation (**high-risk**; with ICH or severe bleeding in ≥1 prior FNAIT pregnancy)
- Have **maternal anti–HPA-1a and/or anti–HPA-5b alloantibodies and an HPA-1a– and/or HPA-5b–positive fetus** in the current pregnancy

Study design

- Participants (N ≈ 50) will be grouped into **2 cohorts based on HPA-1a and HPA-5b incompatibility**
- Within each cohort, participants will be randomly assigned in a 4:1 ratio to receive weekly **intravenous nipocalimab** or weekly **IVIG with prednisone**
- During the treatment period, participants will have **ultrasound monitoring every 2 weeks**
- After birth, babies will receive a cranial ultrasound scan and a platelet count assessment
- Follow-up will be 6 months after delivery for maternal participants and 2 years for babies
- Study sites are located in Austria, Germany, Poland, The Netherlands, the United Kingdom, and the United States

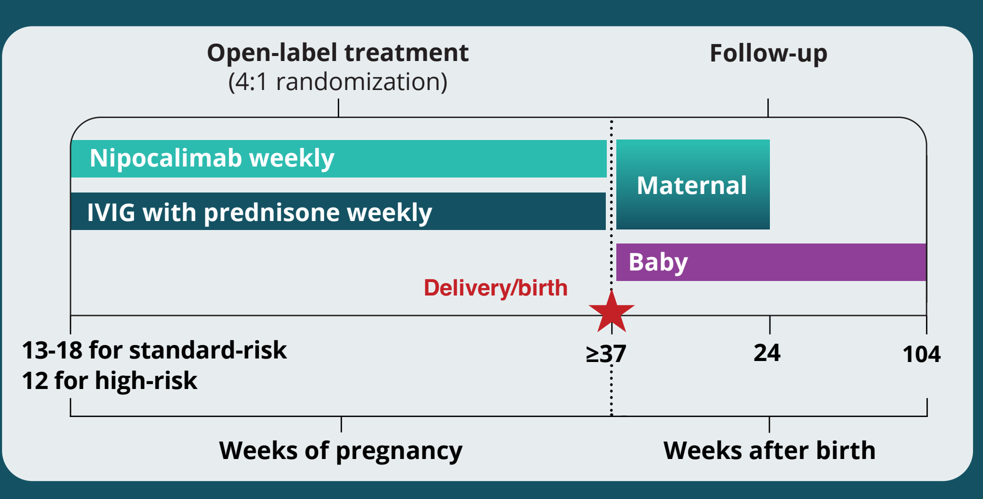

Both studies will evaluate the safety and efficacy of nipocalimab using similar assessments.

Study assessments

Primary composite endpoint:

- Number of fetuses or babies who do not survive, have severe bleeding, or are born with a very low number of platelets

Key secondary endpoints:

- Platelet count of babies at birth
- Number of fetuses or babies who do not survive
- Lowest platelet count level of babies during the first week after birth
- Number of babies who require platelet transfusion(s) or IVIG
- Number of bleeding events in a fetus during pregnancy or during the first week after birth in a baby
- Safety outcomes in maternal participants and babies

Other assessments

- Laboratory parameters
- Pharmacokinetics and pharmacodynamics
- Patient- and caregiver-reported outcomes

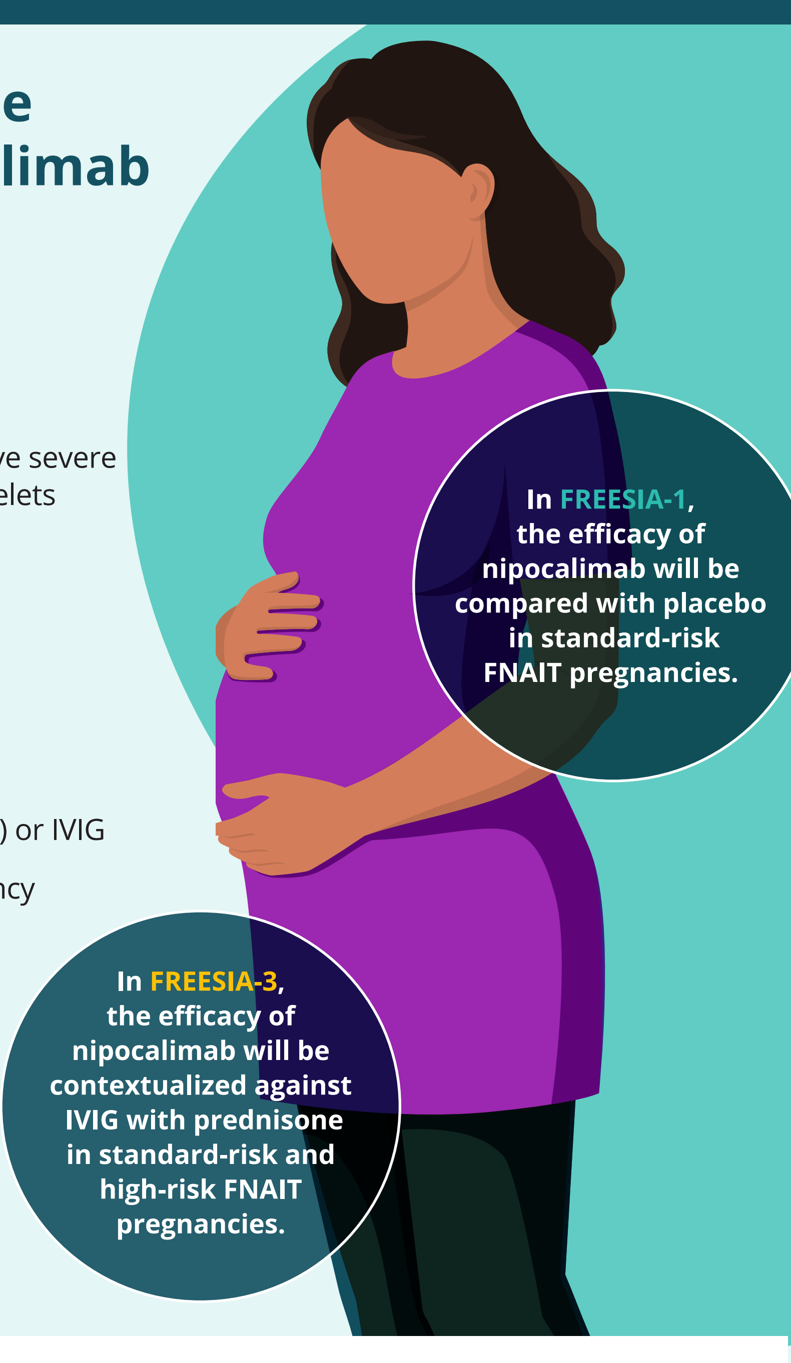

*This figure was reproduced from Komatsu Y, et al. Design of the Phase 3 AZALEA Trial of Nipocalimab in Severe Hemolytic Disease of the Fetus and Newborn. *Am J Perinatol*. 2025;42(7):955-961, in accordance with the terms of the Creative Commons Attribution License (https://creativecommons.org/licenses/by/4.0/).
